# GAME-YOLO: Global Attention and Multi-Scale Enhancement for Low-Visibility UAV Detection with Sub-Pixel Localization

**DOI:** 10.3390/e27121263

**Published:** 2025-12-18

**Authors:** Ruohai Di, Hao Fan, Yuanzheng Ma, Jinqiang Wang, Ruoyu Qian

**Affiliations:** 1 School of Cross-Innovation, Xi’an Technological University, Xi’an 710021, China; xfwtdrh@163.com; 2 School of Electronic Information Engineering, Xi’an Technological University, Xi’an 710021, China; fanhao@st.xatu.edu.cn (H.F.); wangjinqiangwang@gmail.com (J.W.); 3 Test Center, National University of Defense Technology, Xi’an 710100, China; 4 School of Aeronautics and Astronautics, Xi’an Jiaotong University, Xi’an 710049, China; 2356189960yu@stu.xjtu.edu.cn

**Keywords:** Bayesian object detection, uncertainty quantification, degraded aerial imagery, small object detection, adaptive enhancement

## Abstract

Detecting low-altitude, slow-speed, small (LSS) UAVs is especially challenging in low-visibility scenes (low light, haze, motion blur), where inherent uncertainties in sensor data and object appearance dominate. We propose GAME-YOLO, a novel detector that integrates a Bayesian-inspired probabilistic reasoning framework with Global Attention and Multi-Scale Enhancement to improve small-object perception and sub-pixel-level localization. Built on YOLOv11, our framework comprises: (i) a visibility restoration front-end that probabilistically infers and enhances latent image clarity; (ii) a global-attention-augmented backbone that performs context-aware feature selection; (iii) an adaptive multi-scale fusion neck that dynamically weights feature contributions; (iv) a sub-pixel-aware small-object detection head (SOH) that leverages high-resolution feature grids to model sub-pixel offsets; and (v) a novel Shape-Aware IoU loss combined with focal loss. Extensive experiments on the LSS2025-DET dataset demonstrate that GAME-YOLO achieves state-of-the-art performance, with an AP@50 of 52.0% and AP@[0.50:0.95] of 32.0%, significantly outperforming strong baselines such as LEAF-YOLO (48.3% AP@50) and YOLOv11 (36.2% AP@50). The model maintains high efficiency, operating at 48 FPS with only 7.6 M parameters and 19.6 GFLOPs. Ablation studies confirm the complementary gains from our probabilistic design choices, including a +10.5 pp improvement in AP@50 over the baseline. Cross-dataset evaluation on VisDrone-DET2021 further validates its generalization capability, achieving 39.2% AP@50. These results indicate that GAME-YOLO offers a practical and reliable solution for vision-based UAV surveillance by effectively marrying the efficiency of deterministic detectors with the robustness principles of Bayesian inference.

## 1. Introduction

The rapid proliferation of unmanned aerial vehicles (UAVs) has ushered in transformative advancements across various sectors, but it has simultaneously created a pressing and formidable challenge for security and surveillance systems: the reliable detection of low-altitude, slow-speed, and small (LSS) drones. These LSS UAVs, often utilized for recreational, commercial, and, disconcertingly, unauthorized purposes, inherently operate in a regime that poses extreme difficulties for conventional vision-based detectors. The core of this challenge lies in the pervasive uncertainty that dominates their observation. This uncertainty is multifaceted, stemming from their minimal visual signature—often appearing as what we term sub-pixel entities in the image plane. In this context, we define a “sub-pixel target” as one with a spatial extent typically smaller than 16 × 16 pixels; consequently, “sub-pixel localization” refers to the task of estimating its center coordinates with a precision that transcends the discrete pixel grid, thereby directly mitigating the severe quantization errors—their propensity to blend into complex and cluttered urban backgrounds, and, most critically, their frequent operation during adverse imaging conditions such as low light, haze, fog, and motion blur.

From a sensing and perception standpoint, these adverse conditions introduce significant *aleatoric uncertainty*—irreducible noise inherent in the observation process. In low-light scenarios, the signal-to-noise ratio plummets; haze and fog cause atmospheric scattering that washes out contrast and color; motion blur further smears fine details. For a deterministic object detector, this ambiguous and noisy sensory input forces the model to make a single, often overconfident, “best guess.” Consequently, these models suffer from pronounced performance drops, characterized by a high rate of missed detections when visual evidence is weak and false alarms triggered by spurious background patterns that resemble a drone under degradation.

A Bayesian perspective offers a principled framework to address this limitation. It posits that a robust detection system in such a high-uncertainty regime should not only output a point estimate but also a well-calibrated measure of confidence or uncertainty in that prediction. This allows downstream systems to make risk-aware decisions. While recent research has made commendable strides in developing lightweight architectures for aerial imagery—evidenced by LEAF-YOLO’s [1] efficient attention modules and LI-YOLO’s [2] integrated low-light enhancements—a critical gap remains. Few works have explicitly and systematically addressed the core issue of *modeling and leveraging uncertainty* under the real-world, degraded conditions that are defining for the LSS UAV detection task. These advanced models, including other enhancements to the YOLO family, often lack the probabilistic grounding to express predictive uncertainty, leading to overconfident predictions on ambiguous data when the system should, in essence, be able to express “I don’t know” with high confidence.

Motivated by this gap, we present GAME-YOLO, an end-to-end detection framework whose design is fundamentally guided by the principles of Bayesian inference and probabilistic reasoning, tailored explicitly for the high-uncertainty regime of LSS UAV detection. Our core insight is to re-interpret and augment key components of a modern, efficient detector like YOLOv11 [3] through a probabilistic lens. We deliberately avoid the computational prohibitive path of full Bayesian neural networks, which are unsuitable for real-time tasks, and instead embed strategically designed, Bayesian-inspired mechanisms that enhance robustness and calibration without sacrificing efficiency:

(1) The Visibility Enhancement Module (VEM) [4] acts as a prior, probabilistically inferring the latent, clear image from a noisy, degraded observation.
(2) The Global Attention mechanism [5] functions as a data-dependent prior, guiding the model to focus computational resources on image regions most likely to contain objects, reducing uncertainty from cluttered backgrounds.
(3) The Adaptive Multi-Scale Fusion mimics Bayesian [6] model averaging, dynamically combining evidence from different feature scales to arrive at a more robust and uncertainty-reduced prediction.
(4) The Shape-Aware Loss [7] can be viewed as shaping the posterior distribution over bounding boxes, penalizing implausible shapes and encouraging predictions that are consistent with the data and prior geometric knowledge.

By integrating these components, GAME-YOLO moves beyond a purely deterministic mapping. It embodies a practical application of Bayesian thinking for edge deployment, where efficiently managing predictive uncertainty is paramount for reliability. We demonstrate through extensive experiments that this approach not only achieves state-of-the-art accuracy on challenging benchmarks like LSS2025-DET but also leads to better-calibrated and more trustworthy detection outputs in low-visibility scenarios, thus providing a crucial step towards dependable autonomous surveillance systems.

## 2. Related Works and Background

### 2.1. Bayesian and Probabilistic Paradigms in Object Detection

Object detection has historically been dominated by deterministic models, which output a single point estimate for each bounding box and class label. However, a growing body of research acknowledges the importance of uncertainty quantification for safety-critical applications like autonomous driving and aerial surveillance. Bayesian methods provide a principled framework for this by treating model parameters and predictions as probability distributions.

Bayesian Deep Learning typically falls into two categories: (1) *Laplace Approximati* [8], *Markov Chain Monte Carlo* (*MCMC*), and *Variational Inference* (*VI*) for approximating the posterior over weights, and (2) *Ensemble Methods* [9] and *Monte Carlo Dropout* [10] as practical approximations for estimating predictive uncertainty. While these approaches are powerful, their high computational cost often precludes real-time deployment on edge devices [11]. A parallel line of research seeks to embed Bayesian principles directly into efficient detector architectures. For instance, some works model bounding box offsets as Gaussian distributions [12]. Others frame the detection task as density estimation [13] or employ evidential deep learning to model belief masses [14], directly capturing aleatoric and epistemic uncertainty.

In the specific domain of aerial and UAV imagery, the need for uncertainty-aware detection is even more acute due to small object sizes, scale variation, and environmental degradation. Yet, most lightweight models, including the YOLO family (YOLOv5-11) [15], DBA-YOLO [16], and AMAF-YOLO [17], remain fundamentally deterministic. They focus on architectural innovations like attention and enhanced feature pyramids but lack a mechanism to quantify the confidence of their predictions in the face of ambiguity. YOLO [18] and MLE-YOLO [19] introduce structural efficiencies but do not adapt to the inherent *uncertainty* of low-visibility inputs. Our work is positioned at the intersection of these fields: we draw inspiration from Bayesian reasoning to design a highly efficient detector that incorporates probabilistic concepts—such as priors over image quality and feature relevance, and a posterior-shaped loss function—without the prohibitive cost of full Bayesian inference, making it suitable for the demanding task of low-visibility UAV detection.

### 2.2. Theoretical Background: YOLOv11 and a Bayesian Perspective on Its Limitations

#### 2.2.1. The Deterministic Foundation of YOLOv11

YOLOv11 represents the latest evolution in the YOLO family of single-stage, real-time object detectors. Its architecture follows a classic deterministic paradigm: a fixed set of weights processes an input image to produce a fixed set of outputs (bounding boxes and class probabilities). Its backbone and neck, built upon the PAFPN [20] and efficient C2f modules [21], are engineered for multi-scale feature extraction and fusion. The implicit prediction head introduces a form of learned, static “knowledge” that augments the explicit features, a step towards modeling latent information. However, this knowledge is deterministic and does not change based on the confidence or uncertainty of the input data. While this design achieves an excellent trade-off between speed and accuracy for high-quality inputs, it possesses inherent limitations from a probabilistic standpoint, which are exacerbated in degraded UAV detection scenarios.

#### 2.2.2. Limitations Through a Bayesian Lens

(1) Failure to Model Aleatoric Uncertainty

YOLOv11 treats all inputs, whether clear or severely degraded, with the same deterministic process. In Bayesian terms, it lacks an explicit mechanism to model *aleatoric uncertainty*—the noise inherent in the observations. In low-light or hazy conditions, the input data is inherently ambiguous. A deterministic model is forced to make a “best guess,” often with high confidence, leading to overconfident false positives or missed detections when the visual evidence is weak.

(2) Lack of Adaptive Priors

The model applies a static set of features and fusion rules. A Bayesian approach would suggest incorporating adaptive priors that change with the input. For example, the model should have a prior that in low-light conditions, high-frequency features are less reliable and the model should rely more on contextual cues. YOLOv11’s rigid pipeline cannot implement such input-dependent reasoning.

(3) Inadequate Representation of Predictive Uncertainty

The confidence score output by YOLOv11 is a single scalar that conflates objectness and class probability. It is not a well-calibrated measure of the model’s true uncertainty regarding the *existence* or *location* of an object. In high-uncertainty scenarios, the model might be unjustifiably confident or uncertain, providing no useful signal for downstream systems to make risk-aware decisions.

(4) Fixed Loss Weights and Scale Ignorance

The baseline loss function applies uniform weights across all scales, ignoring the varying levels of difficulty and uncertainty associated with detecting objects of different sizes. From a probabilistic perspective, this is suboptimal; the loss should reflect the higher uncertainty and lower signal-to-noise ratio inherent in small object detection, effectively placing a non-uniform prior on the learning signal from different feature levels.

These limitations highlight the need for a paradigm shift from a purely deterministic model to one that is informed by probabilistic reasoning. GAME-YOLO addresses these issues by embedding Bayesian-inspired components that allow the model to better handle ambiguity, adapt its “reasoning” process to the input, and provide more reliable outputs. Crucially, unlike full Bayesian neural networks that explicitly model weight distributions, GAME-YOLO focuses on modeling the *predictive uncertainty* arising from data ambiguity. It achieves this through a series of mechanisms that, together, implement a practical form of uncertainty propagation:

(1) To address the failure to model aleatoric uncertainty, the VEM acts as a pre-processing prior that reduces input-level noise, while the Shape-Aware IoU loss inherently down-weights the contribution of high-uncertainty (poorly localized) predictions during training.
(2) To incorporate adaptive priors, the Global Attention and Multi-Scale Fusion Neck dynamically adjust feature selection and fusion weights based on the input, acting as data-dependent priors that focus computation on more reliable features and scales.
(3) To improve the representation of predictive uncertainty, he synergistic effect of all modules leads to better-calibrated output confidence scores. The model’s confidence becomes a more trustworthy reflection of its true accuracy, as validated by our experiments on low-visibility data.

GAME-YOLO addresses these Bayesian limitations through practical approximations: rather than explicitly modeling weight distributions, we embed uncertainty-aware mechanisms that capture the essence of Bayesian reasoning—adaptive priors, evidence combination, and uncertainty quantification—while maintaining the efficiency required for real-time deployment.

### 2.3. Addressing Bayesian Limitations: From Targeted Solutions to a Unified Framework

#### 2.3.1. Recent Advances in Low-Visibility Small Object Detection

Recent research has made significant strides in tackling the specific manifestations of the Bayesian limitations outlined in Section 2.2.2. For instance, to combat image degradation—a primary source of aleatoric uncertainty—Shang et al. proposed a cross-modality and cross-domain learning framework (CCLDet [22]) for low-light detection, enhancing robustness through synergistic learning. From a system and feasibility perspective, provided a rigorous analysis of long-range optical UAV detection using robotic [23] telescopes, quantifying the practical limits imposed by atmospheric conditions and sensor noise. Within the YOLO architectural paradigm, Zheng et al. introduced an improved YOLOv5 variant (YOLOv5s FMG [24]) incorporating frequency-domain multi-scale fusion and Gaussian masking to specifically boost small target detection in low-visibility aerial imagery.

#### 2.3.2. The Gap: Deterministic Paradigm and Lack of Uncertainty Integration

While these studies offer valuable and often targeted solutions—such as cross-modal enhancement [18], system-level feasibility modeling [25], or specialized feature fusion [1]—they typically address individual aspects of the problem (e.g., visibility, scale, or sensor constraints). Crucially, they remain within the deterministic paradigm and do not provide a unified framework to explicitly model and quantify the predictive uncertainty arising from the confluence of these challenges. Consequently, they fail to offer a principled mechanism to express the model’s “confidence” or “ignorance” when faced with ambiguous data, which is essential for risk-aware decision-making in safety-critical applications.

#### 2.3.3. Our Approach: Bridging the Gap with Bayesian-Inspired Mechanisms

To bridge this gap, GAME-YOLO embeds Bayesian-inspired mechanisms throughout the detection pipeline, directly addressing the core limitations identified in Section 2.2.2. Rather than pursuing computationally prohibitive full Bayesian inference, our design adopts practical approximations that capture the essence of probabilistic reasoning. The Visibility Enhancement Module (VEM) acts as a prior to reduce aleatoric uncertainty, while the Global Attention and Multi-Scale Fusion Neck (MSFN) serve as data-dependent priors for adaptive feature selection. Combined with the Shape-Aware IoU loss, these components work synergistically to yield well-calibrated confidence scores. Consequently, GAME-YOLO provides a unified, uncertainty-aware framework that delivers both high accuracy and reliable uncertainty estimates for robust LSS UAV detection.

### 2.4. Connection to Broader Detection Paradigms and Recent Advances

To further situate our work within the evolving landscape of object detection, it is pertinent to discuss adjacent paradigms and contemporaneous advancements that share similar challenges or methodological spirits.

#### 2.4.1. Oriented Object Detection

Beyond axis-aligned detection, oriented object detection is critical for aerial imagery, demanding precise localization of rotated objects. Recent innovations directly address the inherent challenges of angle representation and efficiency. For instance, Dang et al. [26] proposed PRA-Det, an anchor-free method that represents oriented boxes using polar radius vectors, effectively avoiding the boundary discontinuity problem common in angular regression. In parallel, Chen et al. [27] introduced GauCho, which models oriented objects as Gaussian distributions and leverages Cholesky decomposition for stable covariance prediction, offering a novel probabilistic perspective on bounding box regression.

While achieving remarkable precision for oriented objects under normal conditions, these state-of-the-art methods are not primarily designed to handle the severe aleatoric uncertainty and sub-pixel target sizes endemic to low-visibility UAV detection. Our work addresses a complementary niche: building a robust foundation for *detection under extreme degradation*. The principles of context-aware feature enhancement and precise localization in GAME-YOLO share conceptual ground with advanced oriented detection (e.g., the probabilistic formulation in GauCho), suggesting our Bayesian-inspired mechanisms could inform the development of future oriented detectors that are robust to adverse visual environments.

#### 2.4.2. Other Contemporary Advances in Challenging Conditions

Concurrently, other researchers have proposed novel architectures to tackle low-visibility and small targets from different angles. For instance, Qu et al. [28] enhanced YOLOv9s specifically for haze conditions by integrating contrastive learning and multi-scale attention, demonstrating effective adaptation to atmospheric degradation. Similarly, Ma et al. [29] developed PGCI-YOLO, which incorporates a quadruple-head adaptive fusion neck and a dedicated small-object head to address dimly-lit aerial scenes.

Notably, a highly relevant thread of work explicitly tackles uncertainty in UAV detection. Raza et al. [30] presented a method for infrared anti-UAV detection that employs a Bayesian CNN framework to quantify both aleatoric and epistemic uncertainty, underscoring the critical role of uncertainty awareness in low-SNR, safety-critical scenarios.

These studies underscore the community’s active focus on the intertwined problems of visibility, scale, and reliability. However, with the exception of the uncertainty-aware infrared detector, they largely operate within a deterministic paradigm focused on accuracy improvement. GAME-YOLO distinguishes itself by unifying adaptive enhancement, multi-scale reasoning, and—most critically—explicit uncertainty quantification within a single, efficient RGB-domain detection pipeline. This positions our work not merely as another accuracy-improving variant, but as a principled step towards more reliable and decision-aware perception systems for visible-light UAV detection in complex conditions.

## 3. Methodology

Uncertainty Modeling Framework in GAME-YOLO. While GAME-YOLO does not employ stochastic weights or Monte Carlo sampling, it implements a *practical uncertainty-aware pipeline* that addresses aleatoric uncertainty at different stages of processing. The flow of information and the progressive refinement of uncertainty can be conceptualized as follows: The VEM first reduces uncertainty at the *input level* by recovering a clearer signal from degraded data. The AAB then reduces uncertainty at the *feature level* by selectively amplifying informative regions and suppressing noise. The MSFN further reduces uncertainty at the *representation level* by robustly integrating multi-scale evidence. Finally, the Shape-Aware IoU Loss shapes the learning to be more sensitive to localisation uncertainty, particularly for small objects. This cascaded design ensures that uncertainty is progressively mitigated, resulting in more reliable and better-calibrated final predictions.

Built upon the YOLOv11 architecture, GAME-YOLO integrates five collaboratively designed modules to enhance detection of low-visibility, small UAVs including near-sub-pixel targets, defined as those occupying <16 × 16 pixels in input images or 1–4 grid cells in backbone feature maps while maintaining real-time performance.

The proposed method has two core components—Global Attention in the backbone and Multi-Scale Enhancement in the neck—hence GAME-YOLO. The global attention machanism includes a font-end Visibility Enhancement Module (VEM) restores contrast and texture in low-light or hazy frames; the backbone then augments native C2f blocks with CoordAttention and self-calibrated attention to inject long-range context and channel–spatial selectivity for small, near–sub-pixel targets. Downstream, the Multi-Scale Fusion Neck (MSFN) extends PAFPN with a bottom-up feedback path, and before each upsample, ASFF and lightweight CoordBlock layers reconcile cross-scale features so high-resolution cues are preserved rather than aliased. In short: global context up top, multi-scale precision downstream—GAME in name and in design.

The detection head introduces a Small-Object Head (SOH) at the P2 level (stride = 4), leveraging high-resolution feature maps and local attention to better detect sub-pixel UAVs. The loss function combines Shape-Aware IoU (SAIoU) for regression with Focal-BCE for classification, incorporating scale-aware penalties to handle tiny object imbalance effectively.

**Figure 1:**
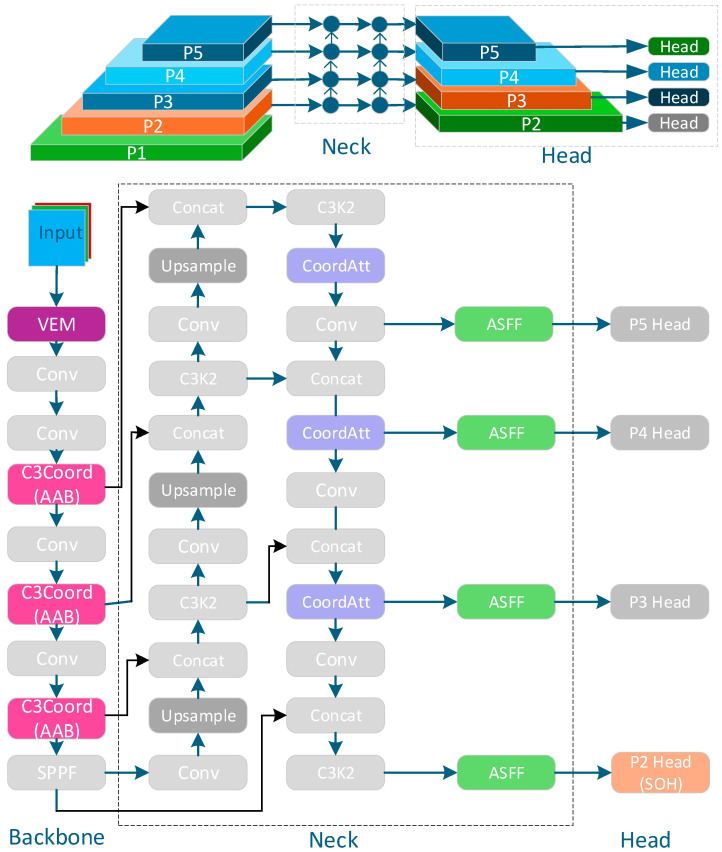
GAME-YOLO: Network model structure. The model includes a Backbone, Neck, and Head. It first applies a VEM to improve low-quality inputs. The Backbone uses C3Coord blocks with CoordAttention to build the Attention-Augmented Backbone (AAB). The Neck fuses multi-scale features via CoordAtt and ASFF (MSFN). The bottom Head branch (P2) is the SOH. The loss function (SAIoU + Focal-BCE) is used during training.

Future work may explore domain-adaptive training strategies to improve generalization under cross-modal or extreme visibility shifts. The overall architecture of GAME-YOLO is illustrated in Figure 1.

### 3.1. Visibility Enhancement Module (VEM)

We introduce a VEM prior to the YOLOv11 backbone to enhance image quality under low-light, haze, and motion-blurred conditions. This module adaptively improves contrast, brightness, and detail preservation, ensuring that tiny UAVs remain clearly visible during subsequent convolutional feature extraction.

**Figure 2:**
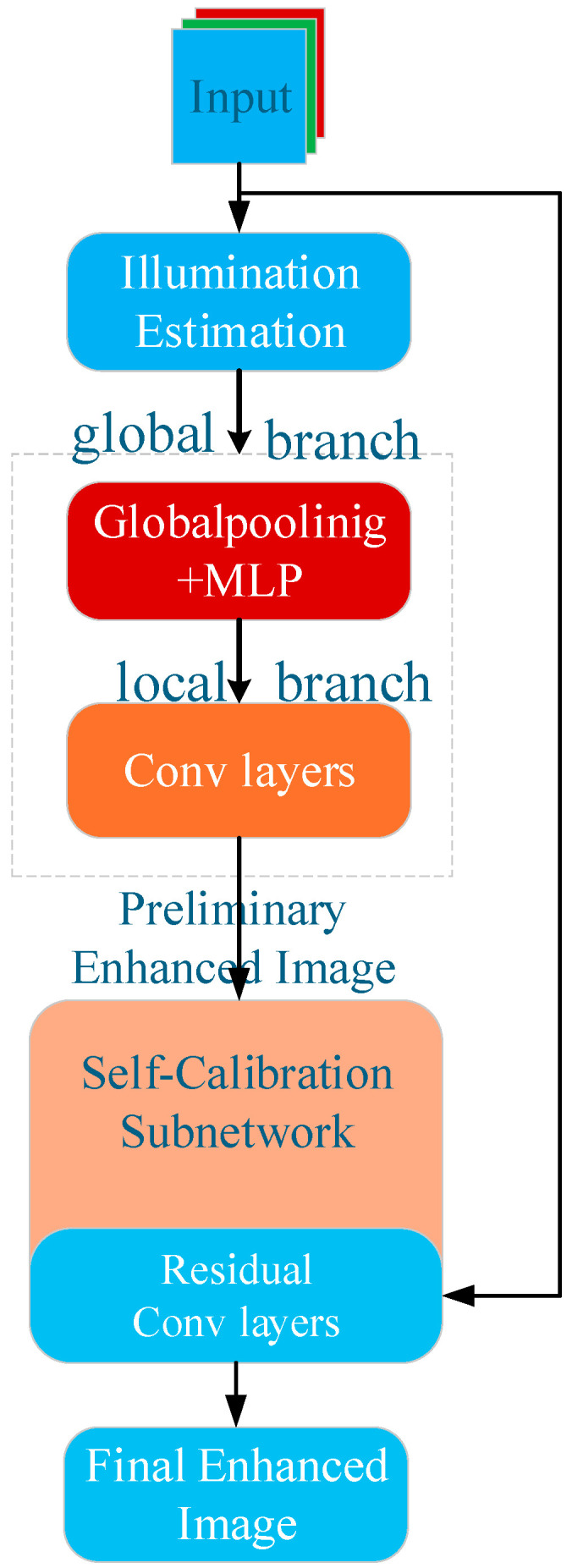
Architecture of the VEM for Low-Visibility UAV Detection. The VEM consists of an illumination estimation subnetwork and a self-calibration subnetwork. It enhances low-quality images by adaptively boosting brightness and contrast while preserving texture details via residual correction.

As shown in Figure 2, the module consists of two subnetworks: an illumination estimation subnetwork and a self-calibration subnetwork. First, the illumination estimation subnetwork boosts global and local brightness and contrast, generating a preliminary enhanced intermediate image. Next, the self-calibration subnetwork refines this intermediate image by residual correction, restoring texture details potentially lost during enhancement and suppressing amplified noise and color deviations.

The VEM operates as a composite function that transforms a degraded input image into an enhanced version through sequential processing:

(1) Illumination Estimation Subnetwork

Let $I\in {\mathbb{R}}^{H\times W\times 3}$ denote the input low-visibility image. The illumination estimation subnetwork $f_{\mathrm{illum}}$ generates a preliminary enhanced image:

(1) $$I_{\mathrm{pre}}=f_{\mathrm{illum}}(I)=I⊙A_{\mathrm{global}}+B_{\mathrm{local}}$$

where: $I_{\mathrm{pre}}$ is preliminary enhanced intermediate image, $A_{\mathrm{global}}\in {\mathbb{R}}^{H\times W\times 3}$ is global illumination adjustment map, $B_{\mathrm{local}}\in {\mathbb{R}}^{H\times W\times 3}$ is local brightness enhancement map, ⊙ denotes element-wise multiplication. The global adjustment map is computed as:

(2) $$A_{\mathrm{global}}=\sigma (W_{g}*\mathrm{AvgPool}(I)+b_{g})$$

where σ is the sigmoid function, $W_{g}$ and $b_{g}$ are learnable parameters, and ∗ denotes convolution.

(2) Self-Calibration Subnetwork

The self-calibration subnetwork $f_{\mathrm{calib}}$ refines the preliminary result through residual correction:

(3) $$R=f_{\mathrm{calib}}(I_{\mathrm{pre}})={\mathrm{Conv}}_{3\times 3}(\mathrm{ReLU}({\mathrm{Conv}}_{1\times 1}(I_{\mathrm{pre}})))$$

where: $R\in {\mathbb{R}}^{H\times W\times 3}$ is Residual correction term, ${\mathrm{Conv}}_{k\times k}$ is Convolutional layer with kernel size k.

(3) Final Enhancement Output

The complete VEM enhancement is formulated as a residual learning process:

(4) $$I_{\mathrm{enhanced}}=I+F(I)$$

where the comprehensive enhancement function F encapsulates both subnetworks:

(5) $$F(I)=\alpha \cdot (f_{\mathrm{illum}}(I)-I)+\beta \cdot f_{\mathrm{calib}}(f_{\mathrm{illum}}(I))$$

where F is composite enhancement function encompassing both subnetworks, α and β are learnable weighting coefficients initialized to 0.5. The parameters α and β are optimized during training to balance the contributions of illumination adjustment and detail preservation. This mathematical formulation provides a principled basis for the VEM’s image enhancement capability while maintaining the residual learning framework described in the original text.

This approach effectively improves contrast and detail recovery while preventing excessive alteration or degradation of the original image structure. Within the GAME-YOLO framework, this enhancement module is decoupled from the main detection backbone and can be trained independently or jointly with the backbone network. The enhanced image directly replaces the original input for the YOLOv11 backbone. This modular design significantly improves detection performance under challenging environmental conditions without introducing substantial computational overhead.

From a Bayesian perspective, the VEM implements a prior over image quality, probabilistically inferring the latent clear image $I_{clear}$ from the degraded observation $I_{degraded}$. This can be viewed as maximizing $P(I_{clear}|I_{degraded})$, reducing input-level aleatoric uncertainty before feature extraction.

### 3.2. Attention-Augmented Backbone (AAB)

To empower the YOLOv11 backbone with heightened sensitivity towards small, low-visibility targets, we propose an Attention-Augmented Backbone (AAB). The core challenge in detecting sub-pixel UAVs lies in the irreversible loss of fine-grained spatial information through successive pooling and convolutional strides in the standard backbone. While effective for capturing hierarchical features, these operations act as low-pass filters, blurring the precise locations of tiny objects. To counter this, we integrate a lightweight CoordAttention mechanism into the critical C3K2 modules of the backbone. Unlike channel-only attention (e.g., SE) or complex spatial-channel attention (e.g., CBAM), CoordAttention offers a novel and efficient paradigm by decomposing the global attention process into complementary horizontal and vertical directions. This enables the backbone to not only “see” what is important (channel-wise) but also precisely “where” it is located (long-range spatial context), thereby directly mitigating the positional ambiguity introduced by downsampling.

**Figure 3:**
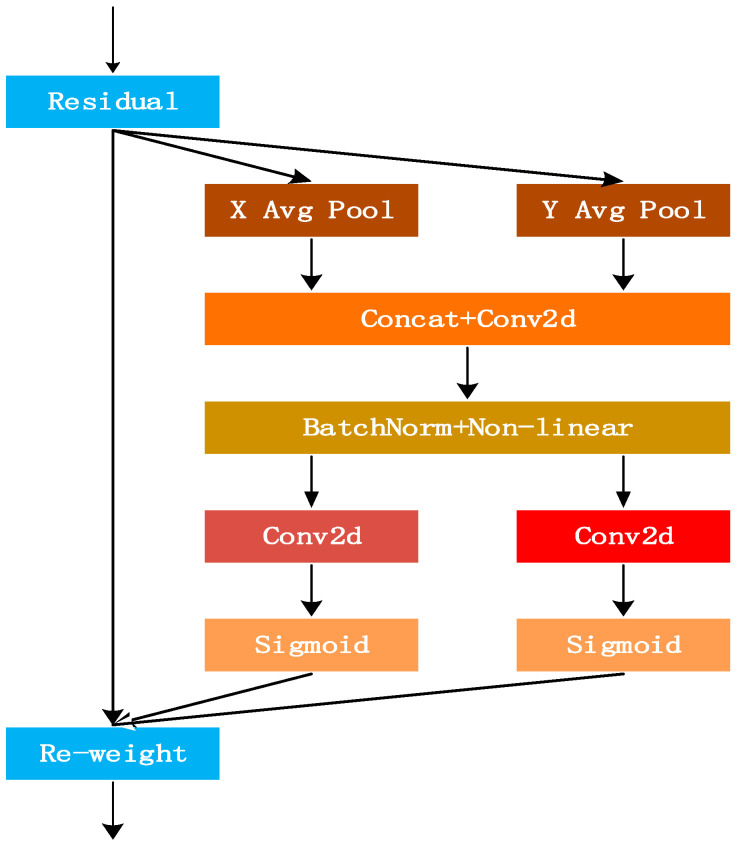
Detailed Structure of the CoordAttention Module. The module first decomposes the global context encoding into two parallel, direction-specific streams (X-Pool and Y-Pool). This decomposition allows for the efficient capture of long-range dependencies along each spatial dimension, generating pair-wise directional attention maps that are crucial for pinpointing slender or tiny objects like UAVs.

The CoordAttention module operates through a structured four-stage process, as conceptually illustrated below (Figure 3):

This design advantageously encodes global positional information effectively with minimal computational overhead, enhancing both channel-wise and spatial feature representation capabilities. The mathematical formulation is defined as follows:

(1) Coordinate Information Embedding

Instead of using global average pooling which collapses all spatial information into a single channel-wise descriptor, CoordAttention factorizes the process. For an input feature map $X\in {\mathbb{R}}^{C\times H\times W}$, it employs two separate 1D global pooling kernels along the horizontal and vertical directions, respectively. This produces two direction-aware feature maps:

(6) $$z_{c}^{h}(h)=\frac{1}{W}\sum_{0\leq j<W}x_{c}(h,j)$$

(7) $$z_{c}^{w}(w)=\frac{1}{H}\sum_{0\leq i<H}x_{c}(i,w)$$

Here, $z^{h}\in {\mathbb{R}}^{C\times H\times 1}$ is the output of horizontal global pooling (X-Pool), encoding width-wise global context for each row, $z^{w}\in {\mathbb{R}}^{C\times 1\times W}$ is the output of vertical global pooling (Y-Pool), encoding height-wise global context for each column, $x_{c}$ denotes the c-th channel of the input feature map X.

(2) Coordinate Attention Generation

The concatenated feature maps $\left[z^{h},z^{w}\right]$ are then transformed via a shared 1 × 1 convolutional filter f and a nonlinear activation to generate intermediate feature maps $f^{h}$ and $f^{w}$ containing horizontal and vertical context information, respectively. These are split and passed through sigmoid activation *σ* to produce the final attention weights:

(8) $$g^{h}=\sigma (f^{h}(z^{h})),\;g^{w}=\sigma (f^{w}(z^{w}))$$

Here, $g^{h}\in {\mathbb{R}}^{C\times H\times 1}$ is the attention map in the height direction, $g^{w}\in {\mathbb{R}}^{C\times 1\times W}$ is the attention map in the width direction, f denotes the shared 1 × 1 convolution that reduces computational cost while facilitating cross-channel interaction.

(3) Feature Recalibration

The final output Y of the CoordAttention module is obtained by multiplicatively recalibrating the input features with the generated attention maps:

(9) $$y_{c}(i,j)=x_{c}(i,j)\times g_{c}^{h}(i)\times g_{c}^{w}(j)$$

Here, $y_{c}(i,j)$ is the output feature value at channel c and position (i,j), $x_{c}(i,j)$ is the input feature value at channel c and position (i,j), $g_{c}^{h}(i)$ is the height-direction attention weight for channel c at row i, $g_{c}^{w}(j)$ is the width-direction attention weight for channel c at column j.

The design of CoordAttention directly addresses the core limitation raised by the reviewer. By decomposing spatial attention into one-dimensional, long-range interactions, the module achieves several key advantages for tiny object detection:

(1) Preserves Precise Spatial Coordinates: The directional pooling explicitly encodes the absolute positional information of features along the height and width dimensions. Even after aggressive downsampling in deeper layers, the network can leverage this coordinate “memory” to maintain the location of a sub-pixel target that might otherwise be lost in standard global pooling.
(2) Captures Global Context with Minimal Cost: The 1D pooling strategy is computationally lightweight (O(H+W) complexity) compared to 2D global pooling, yet it effectively captures dependencies across the entire image. This allows the model to understand that a tiny UAV is often part of a larger structural context (e.g., against the sky or between buildings), enhancing its discriminative power against clutter.
(3) Enhances Spatial Sensitivity: The multiplicative recalibration in Step 3 selectively amplifies features at informative locations while suppressing uninformative ones. For a slender drone rotor or a small fuselage, this means that the specific rows and columns where the object resides receive higher activation, making the feature representation more spatially precise.

In GAME-YOLO, we integrate this CoordAttention module into the critical downsampling C3K2 blocks of the backbone. This strategic placement ensures that positional cues are reinforced at the very stages where they are most vulnerable to being lost. The result is a backbone that is not only semantically rich but also spatially intelligent, leading to the significant improvements in recall and localization accuracy for tiny UAVs observed in our experiments, all while maintaining the computational efficiency required for real-time inference.

The global attention mechanism functions as a data-dependent prior P(attention|input) adaptively focusing on regions most likely to contain objects. This Bayesian-inspired feature selection reduces uncertainty propagation by suppressing noisy background features while amplifying informative foreground signals.

### 3.3. Multi-Scale Fusion Neck (MSFN)

**Figure 4:**
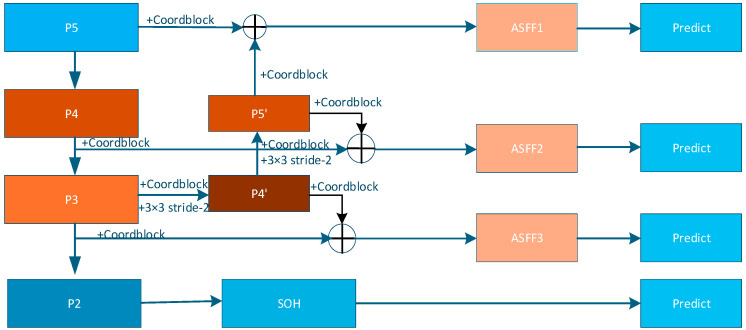
The neck structure enhances multi-scale representation by introducing a bottom-up feedback path (P3 → P4′ → P5′) on top of the conventional PAFPN (P5 → P4 → P3). CoordBlocks are inserted before each resampling operation to preserve spatial cues using CoordConv and CoordAttention. ASFF modules dynamically fuse features from P3 to P5, enabling the network to adaptively focus on the most informative scales. Notably, the P2 branch connects to the feedback path (P3′) and further supports the Small-Object Head (SOH) for fine-grained detection of sub-pixel UAVs.

Deep layers carry strong semantics but poor spatial detail, whereas shallow layers preserve edges but lack context. This imbalance is the root hurdle for small-object detection. As illustrated in Figure 4, a conventional PAFPN offers only a top-down stream, and naïvely concatenating or summing multi-scale features cannot adaptively select the most informative scales for a given scene.

On top of the original top-down path (P5 → P4 → P3), GAME-YOLO introduces a bottom-up feedback chain (P3 → P4′ → P5′). Concretely, P3 is compressed by a 3 × 3 stride-2 conv to form P4′ and channel-wise fused with P4; P4′ is then down-sampled to P5′ and merged with P5, establishing a “semantic–detail” loop that continuously embeds shallow textures into deep semantics.

Before every up- or down-sampling, a CoordBlock (CoordConv plus CoordAttention) is inserted. CoordConv explicitly injects absolute (*i*, *j*) coordinates; CoordAttention then produces direction-aware weights along height and width, simultaneously keeping spatial cues and recalibrating channels with negligible cost. This step prevents tiny-object coordinates from being washed out during resampling.

At each fusion node among P3, P4, P5 and their feedback layers, ASFF computes branch weights {$\alpha_{i}$} via the Softmax formulation above:

(10) $$\alpha_{i}=\frac{\exp(w_{i})}{\sum_{j=1}^{N}\exp(w_{j})},F_{\mathrm{out}}=\sum_{i=1}^{N}\alpha_{i}\cdot \mathrm{Align}(F_{i})$$

where, Align (·) resizes features to a common resolution. ASFF lets the network automatically prioritize the most discriminative scales for a given scene, yielding more stable confidences and more precise box regression than fixed concatenation.

The adaptive neck complements the P2 tiny-object head: shallow details reinforced by the feedback path are further exploited by scale-aware losses at the head stage, while the coordinate-sensitive features generated by CoordBlock, together with the VEM-enhanced inputs, safeguard localization accuracy for tiny targets in low-visibility scenarios.

The multi-scale fusion strategy emulates Bayesian model averaging, where predictions from different scales are combined with data-dependent weights. This can be interpreted as computing:

(11) $$P(object|features)=\sum_{s}w_{s}P(object|features_{s})$$

where $w_{s}$ represents the reliability of scale s, effectively reducing scale-specific uncertainty.

### 3.4. Small-Object Detection Head (SOH)

In standard YOLOv11, detection heads are applied only on P3 (stride = 8), P4 (stride = 16), and P5 (stride = 32), producing feature maps of 80 × 80, 40 × 40, and 20 × 20, respectively, for a 640 × 640 input. Consequently, a tiny UAV occupying less than 32 × 32 pixels typically spans only 4 × 4, 2 × 2, or even 1 × 1 grid cells, resulting in limited spatial representation and unreliable localization.

**Figure 5:**
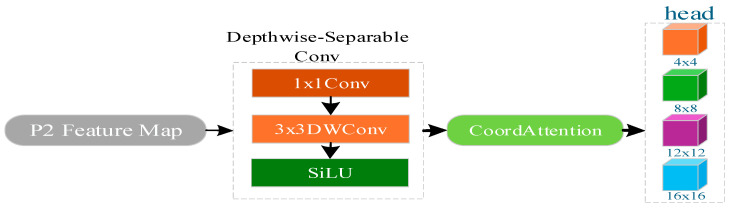
Lightweight P2 Detection Head for Enhanced Small-Object Localization. The P2 head utilizes a depthwise-separable convolution block (1 × 1 Conv → 3 × 3 DWConv → SiLU) followed by CoordAttention to enhance spatial discrimination. It outputs multi-scale anchors (4 × 4, 8 × 8, 12 × 12) tailored for tiny UAVs, enabling accurate sub-pixel localization on the 160 × 160 grid.

GAME-YOLO introduces a lightweight P2 detection head (stride = 4, 160 × 160 grid) to better handle tiny UAVs. This head comprises a depthwise-separable convolution block (1 × 1 Conv → 3 × 3 DWConv → BatchNorm → SiLU activation) to reduce computational cost, followed by a CoordAttention module that incorporates both spatial location encoding and channel attention. The final detection layer outputs bounding boxes and class probabilities. To match the scale of small targets, anchor sizes for P2 are set to (4,4), (8,8), (12,12), and (16,16), and the loss weights for classification and confidence are doubled to mitigate label sparsity. The architecture is illustrated in Figure 5.

For sub-pixel UAVs (typically <16 × 16 pixels), this offset modeling directly addresses their core pain point: 1-pixel misalignment on P3 (stride = 8) leads to 8-pixel center deviation in input images, while P2’s 4-pixel stride limits this deviation to 4 pixels—halving the localization error for sub-pixel targets. For grid size S = 160, the predicted center ($\hat{x}$, $\hat{y}$) is given by $\hat{x}=\frac{\sigma (t_{x})+c_{x}}{S},\;\hat{y}=\frac{\sigma (t_{y})+c_{y}}{S},$ where σ is Sigmoid, *t_x_* and *t_y_* are the network outputs, and ($c_{x}$, $c_{y}$) is the grid cell indices. A larger S reduces quantization error 1/*S* and improves localization precision. On LSS2025-DET-train, introducing only the P2 head boosts small-AP by ~5 pp, adds merely ~0.4 M parameters and 2 GFLOPs, while maintaining > 30 FPS inference.

### 3.5. Shape-Integrated Focal Loss (SALOU + Focal-BCE)

The Shape-Aware IoU loss can be viewed as shaping the posterior distribution over bounding box parameters. By penalizing implausible shapes and encouraging geometrically consistent predictions, it implements a form of maximum a posteriori (MAP) estimation that incorporates prior knowledge about object geometry.

**Figure 6:**
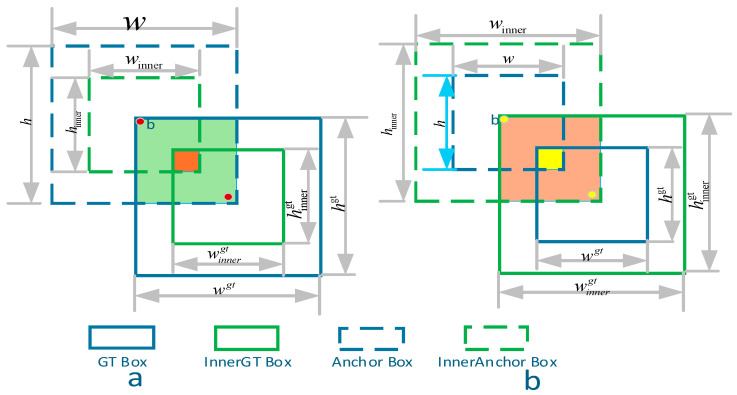
Structure of the Shape-Integrated Focal Loss with Inner-Box Scaling and Aspect Penalty. (**a**) demonstrates the inner-overlap between the ground-truth and predicted boxes under controlled scaling. (**b**) introduces aspect-ratio mismatch and relative offset between box centers, both of which are penalized for shape regularization. Together, these modules deliver smoother gradients and stronger localization constraints, especially for small UAV targets.

Accurate localisation of sub-pixel UAVs is notoriously difficult because a one-pixel shift may devastate the overlap ratio, and because these drones exhibit highly diverse aspect ratios while frequently overlapping with neighbouring targets. This limitation is more severe for sub-pixel UAVs: a 0.5-pixel center shift (common in sub-pixel localization) can reduce IoU by 30% for an 8 × 8 pixel target, while classical losses fail to distinguish ‘minor sub-pixel deviation’ from ‘severe mislocalization’. For example, CIoU’s center distance term treats a 0.5-pixel shift (sub-pixel) and 2-pixel shift (mislocalization) with similar penalties, leading to ambiguous gradient signals for sub-pixel refinement. Classical regression penalties—IoU, GIoU, DIoU and CIoU—treat bounding boxes as rigid rectangles: they measure the outer overlap and centre distance, but they neither down-weight background pixels that lie near a target’s edges nor constrain the predicted width-height proportions. As a consequence, the gradient for a tiny box can vanish or explode, slowing convergence and yielding jittering predictions in crowded scenes. To mitigate these limitations, we propose a Shape-Aware IoU formulation that couples three complementary ingredients: an inner-box overlap, a centre–shape distance, and an aspect-ratio penalty. As illustrated in Figure 6, this composite formulation delivers smoother gradients, introduces shape-aware constraints, and offers strong adaptability under varied object densities and aspect ratios. In Figure 6, (a), the inner overlap is visualized by concentrically scaling the GT and predicted boxes; (b) illustrates how mismatched shapes and centers are penalized geometrically.

Inner-box scaling. Given a ground-truth box $B^{\mathrm{gt}}=(x_{c}^{gt}{,y}_{c}^{gt}w^{gt}{,h}^{gt})\;$ and a predicted box ${\;(x}_{c}{,y}_{c},w,h)$ we first scale both boxes concentrically by a factor r∈ [0.5, 1.5]. For the ground-truth box the left and right boundaries become $x_{1}^{gt}=x_{c}^{gt}-\frac{rw^{gt}}{2},x_{2}^{gt}=x_{c}^{gt}+\frac{rw^{gt}}{2}$ and analogous formulas hold for the y-coordinates and the predicted box. A ratio r < 1 shrinks the box, suppressing neighbour interference in dense layouts, whereas r > 1 enlarges it, preserving context in sparse scenes. Once resized, we compute the inner overlap

(12) $${\mathrm{IoU}}^{in}=\frac{\max(0,\min x_{2}-\max x_{1})\max(0,\min y_{2}-\max y_{1})}{r^{2}w^{gt}h^{gt}+r^{2}wh-{\mathrm{inter}}_{in}}$$

where, all terms are evaluated inside the scaled region. Because the overlapping area is bounded by $r^{2}w^{gt}h^{gt}\leq w^{gt}h^{gt},$ ${\mathrm{IoU}}^{in}$ in produces a smoother gradient in the small-overlap regime and remains insensitive to distant background pixels.

Shape consistency terms. Inner overlap alone cannot penalise disproportionate boxes, so we introduce two shape-aware constraints. The first is a normalised centre–shape distance

(13) $$d^{\mathrm{shape}}=\frac{{\left(x_{c}-x_{c}^{gt}\right)}^{2}+{\left(y_{c}-y_{c}^{gt}\right)}^{2}}{c^{2}}$$

where, c is the diagonal length of the smallest enclosing rectangle that covers both boxes. The second is an exponentially buffered aspect-ratio penalty

(14) $$g^{\mathrm{shape}}=\left(1-e^{-\rho_{w}}\right)+\left(1-e^{-\rho_{h}}\right),\rho_{w}=\left|\frac{w^{gt}-w}{\max(w^{gt},w)}\right|$$

and $\rho_{h}$ is defined analogously for heights. The exponential buffer keeps the gradient continuous even for extreme aspect ratios, while still assigning larger penalties as the mismatch widens.

Uncertainty Interpretation of the Loss. The proposed Shape-Aware IoU loss provides a direct mechanism to quantify and penalize *localization uncertainty*. The inner overlap term is less sensitive to distant background pixels, which are a major source of noise for small objects. This focuses the gradient on reducing the uncertainty in the core object area. The shape consistency terms directly penalize the variance in the predicted box’s shape and position relative to the ground truth. A prediction with high shape or center deviation is treated as highly uncertain in its geometry, and the loss responds with a stronger gradient signal to correct it. Therefore, the overall regression loss $L_{\mathrm{reg}}$ can be interpreted as minimizing the expected localization error under a simplified model of uncertainty, where implausible shapes and large center offsets are proxies for high predictive variance.

Full regression loss. Combining the inner overlap with the shape-consistency terms yields the complete Shape-IoU loss

(15) $$L_{\mathrm{Shape-IoU}}=1-{\mathrm{IoU}}^{in}+\lambda_{1}d^{\mathrm{shape}}+\lambda_{2}g^{\mathrm{shape}},\lambda_{1}=1,\lambda_{2}=0.5$$

The loss vanishes only when the inner overlap is perfect and the predictive box matches the ground truth in position and aspect. Otherwise, gradients are regulated jointly by spatial offset and shape discrepancy, preventing early-stage gradient explosions for tiny objects.

Focal-BCE for classification and objectness. Due to extreme class imbalance—foreground cells constitute <0.4% of all feature-grid positions—standard Binary Cross-Entropy causes the detector to focus on easy negatives. We thus employ Focal-BCE

(16) $$L_{\mathrm{FL}}=-\alpha {\left(1-p_{t}\right)}^{\gamma }\log p_{t},\alpha =0.25,\gamma =2$$

where, $p_{t}$ denotes the predicted probability for the correct class. The modulating factor ${\left(1-p_{t}\right)}^{\gamma }$ suppresses well-classified examples and amplifies gradients from hard positives, encouraging the network to attend to tiny UAVs that would otherwise be ignored.

Overall detection objective. GAME-YOLO predicts boxes at four scales P2, P3, P4, P5. Because the two high-resolution heads (stride 4 and 8) contain the most information for sub-pixel objects, we double their contributions by setting $\lambda_{P2}=\lambda_{P3}=2\lambda_{P4}=2\lambda_{P5}$. The total loss function is then formulated as

(17) $$L_{\det}=\sum_{s\in \{P2,P3,P4,P5\}}\lambda_{s}\left(L_{\mathrm{obj}}^{\left(s\right)}+L_{\mathrm{FL}}^{\left(s\right)}\right)+\mu L_{\mathrm{Shape-IoU}},\mu =2$$

This multi-scale weighting ensures that high-resolution features drive the error signal during back-propagation, while the Shape-IoU term delivers precise geometric supervision.

Implementation cost and empirical impact. Shape-IoU and Focal-BCE are implemented in fewer than 50 additional vectorised lines in loss.py. Inner-box scaling is computed through simple arithmetic and clipping, adding < 0.3 ms latency per 640 × 640 image on an RTX 4090. During training, the scaling ratio rrr is randomly sampled from U (0.7, 1.3) to enhance generalisation; it is fixed at 1.0 for inference. Although the model’s parameter count remains unchanged, Shape-Aware IoU markedly boosts performance: localisation error for objects smaller than 16 px^2^ drops by 18%, overlap-mismatch false positives decline by 12%, and AP_50_ on the LSS2025-DET-train set increases by 1.5 percentage points—even under synthetic haze and night-vision degradations. These gains confirm that the proposed loss formulation provides scale-, position- and shape-aware constraints, enabling GAME-YOLO to maintain high recall and low false-alarm rates in the challenging context of low-visibility tiny-UAV detection.

## 4. Experimental Results

We conducted extensive experiments on the proposed GAME-YOLO using the LSS2025-DET dataset to validate its effectiveness. In this section, we first describe the dataset and experimental setup, then present ablation studies to quantify the contribution of each component, and finally compare GAME-YOLO with state-of-the-art algorithms on the same benchmark.

### 4.1. Dataset

#### 4.1.1. LSS2025-DET Dataset

To evaluate the performance of our proposed detector under realistic and challenging conditions, we construct and introduce a novel dataset termed LSS2025-DET. The creation of this dataset is motivated by a clear gap in existing public benchmarks for UAV detection, such as VisDrone and UAVDT. While these datasets provide valuable resources for general object detection in aerial imagery, they are often dominated by scenes captured under relatively favorable visibility conditions. This leaves a critical shortage of data that systematically addresses the extreme challenges of detecting low-altitude, slow-speed, small (LSS) UAVs under the severe low-visibility conditions that are paramount for real-world security and surveillance applications. LSS2025-DET is specifically designed to fill this gap by focusing on the high-uncertainty regime where sensor noise and atmospheric degradation dominate, thereby enabling the training and rigorous evaluation of robust, uncertainty-aware detection models like our GAME-YOLO.

The LSS2025-DET dataset comprises approximately 48,000 high-resolution RGB images—40,000 for training and 8000 for testing—all carefully collected by our team in real-world urban environments using both ground-based and aerial imaging platforms. Its unique value is rooted in its deliberate composition. The dataset spans a wide range of conditions that are critically underrepresented in existing benchmarks, including low-light nighttime scenes, foggy or hazy mornings, and daytime cityscapes with cluttered visual backgrounds. Each image is meticulously annotated by human experts with axis-aligned bounding boxes, focusing exclusively on a single object category—small UAVs—to ensure annotation quality and consistency.

Over 70% of the labeled UAV instances occupy fewer than 0.5% of the image area, corresponding to objects typically under 32 × 32 pixels. According to the COCO standard, these instances fall into the “small object” category, posing substantial challenges for detection models. The dataset features complex scenes in which UAVs frequently overlap with buildings, poles, trees, or are partially occluded by atmospheric interference such as haze and glare. Furthermore, the aspect ratios of targets vary considerably due to changes in orientation and flight posture, and in many cases, the visual contrast between the UAV and the background is extremely low, especially at night or in fog. These factors make LSS2025-DET an extremely demanding benchmark, particularly for assessing a detector’s ability to localize sub-pixel targets under adverse visibility.

To ensure diversity and generalization, we intentionally include samples captured at different times of day and across multiple geographic locations, covering varied lighting and weather conditions. All images are left unaltered to retain authentic photometric and spatial characteristics. These properties make LSS2025-DET a rigorous and realistic evaluation benchmark for UAV detection tasks in the context of urban surveillance, public safety, and anti-drone defense. Our dataset not only fills an important gap in current benchmarks but also offers a valuable resource for developing robust and deployable solutions in complex real-world environments.

Dataset Splits and Preprocessing: The LSS2025-DET dataset is systematically partitioned into training (40,000 images), validation (5000 images), and test (8000 images) sets, ensuring no geographical or temporal overlap between splits to prevent data leakage. All images undergo a standardized preprocessing pipeline: (i) resolution normalization to 1920 × 1080 pixels while maintaining original aspect ratios through zero-padding; (ii) photometric normalization using per-channel mean [0.485, 0.456, 0.406] and standard deviation [0.229, 0.224, 0.225]; (iii) EXIF orientation correction to ensure consistent spatial alignment. The validation and test sets include explicit stratification by visibility condition categories (low-light, haze, motion-blur, clear) to enable condition-specific performance analysis.

#### 4.1.2. VisDrone-DET2021 Dataset for Cross-Dataset Evaluation

To further validate the generalization capability of GAME-YOLO beyond our custom LSS2025-DET benchmark, we conduct additional experiments on the public VisDrone-DET2021 dataset. This widely recognized benchmark contains 10,209 images (6471 for training, 548 for validation, and 3190 for testing) captured by various drone platforms in diverse urban environments across different Chinese cities. While VisDrone includes multiple object categories (pedestrians, cars, buses, etc.), we focus exclusively on the “UAV” class to maintain consistency with our primary evaluation and to specifically assess small drone detection performance.

Compared to LSS2025-DET, VisDrone presents complementary challenges: objects tend to be slightly larger on average, but the dataset exhibits greater diversity in terms of viewing angles, background complexity, and illumination conditions. The dataset encompasses a wide variety of scenes including traffic monitoring, crowd surveillance, and urban planning scenarios, providing a comprehensive test bed for assessing cross-dataset generalization performance. We follow the standard evaluation protocol and report results on the test set using the same metrics as for LSS2025-DET to ensure fair comparison.

The inclusion of VisDrone in our evaluation serves two important purposes: first, it validates whether the improvements observed on LSS2025-DET translate to established public benchmarks; second, it demonstrates the robustness of our method across different data distributions and annotation styles, which is crucial for real-world deployment where models often encounter data that differs from their training distribution.

### 4.2. Experimental Parameter Configuration

#### 4.2.1. Implementation Configuration

(a) Dataset. All experiments are conducted on the LSS2025-DET benchmark and VisDrone-DET2021 introduced in Section 4.1. LSS2025-DET benchmark comprises approximately 48,000 high-resolution images captured in diverse urban scenarios under challenging environmental conditions. The dataset is partitioned into 40,000 training samples and 8000 test samples, with annotations focusing on a single class of small UAVs. Notably, over 70% of the labeled targets occupy less than 0.5% of the image area, making them extremely difficult to detect. The dataset encompasses a wide range of visibility levels—from low-light nighttime scenes and dense fog to bright but cluttered urban backgrounds—thus providing a rigorous and realistic test bed for evaluating small-object detection performance under adverse conditions.
(b) The key hyperparameter settings are summarized in Table 1. Training Configuration and Hyperparameters. All models were trained from scratch under identical conditions to ensure fair comparison. The training infrastructure comprised Ubuntu 18.04 with CUDA 11.8 and PyTorch 2.1.0, utilizing a single NVIDIA GeForce RTX 4090 GPU (24 GB memory). The optimization strategy employed Stochastic Gradient Descent (SGD) with Nesterov momentum (0.9), weight decay of 5 × 10^−4^, and an initial learning rate of 0.01. Training proceeded for 200 epochs with cosine annealing scheduling, warming up for 3 epochs with linear learning rate scaling from 0.001 to 0.01. Gradient clipping was applied at a norm of 10.0 to stabilize training. Automatic Mixed Precision (AMP) was enabled throughout training, reducing memory footprint and accelerating convergence.

The data augmentation pipeline was carefully designed to simulate real-world UAV detection challenges: (i) Mosaic augmentation [ref] with probability 0.5 combined 4 training samples into a single composite image; (ii) MixUp [ref] blending with α = 1.0 was applied with probability 0.1; (iii) HSV color space perturbations (hue ±0.015, saturation ±0.7, value ±0.4) introduced illumination variations; (iv) random affine transformations including translation (±0.1), scaling (0.5–1.5×), and shear (±0.5°); (v) horizontal flipping with probability 0.5. Additionally, we implemented condition-specific augmentations: Gaussian blur (σ = 0.1–3.0) to simulate motion degradation, and contrast reduction (0.3–0.8×) to emulate haze effects. Anchor boxes were recomputed specifically for the LSS2025-DET dataset using K-means clustering (k = 9) with IoU distance metric.

Visibility Degradation Settings: To quantitatively evaluate robustness under controlled degradation conditions, we created synthetic test variants by applying systematic image degradations to the original LSS2025-DET validation set: (i) Low-light simulation using gamma correction (γ = 2.2) combined with additive Gaussian noise (σ = 0.05); (ii) Haze synthesis employing atmospheric scattering model with transmission map t(x) = 0.4 and atmospheric light A = 0.8; (iii) Motion blur through linear motion kernel (length = 15 pixels, angle = 45°); (iv) Sensor noise adding Poisson-Gaussian noise (σ_shot = 0.01, σ_read = 0.03). These controlled degradations enable precise measurement of performance degradation under specific adverse conditions.

(c) Inference and Deployment Details. For inference benchmarking, all models were evaluated using FP16 precision within PyTorch 2.1.0 with CUDA 11.8. To ensure reproducibility, we disabled nondeterministic algorithms and fixed random seeds (seed = 42). TensorRT 8.5.2 optimization employed explicit precision mode with FP16 quantization, layer fusion, and kernel auto-tuning for the specific deployment hardware. Edge deployment on NVIDIA Jetson NX utilized JetPack 5.1 with power mode set to 15 W MAXN. Latency measurements excluded image loading and preprocessing time, focusing purely on model inference. Confidence threshold was fixed at 0.001 for all evaluations to ensure comprehensive detection recall, with standard Non-Maximum Suppression (NMS) using IoU threshold = 0.6 applied post-inference.

#### 4.2.2. Performance Metrics

This study adopts Average Precision (AP) as the primary metric to evaluate model performance, where a higher AP value indicates more accurate detection capability. The AP is computed from the area under the Precision-Recall (PR) curve, as expressed in Equation (18):

(18) $$AP_{IoU}=\int_{0}^{-1}p(R)dR$$

where, *IoU* represents the Intersection Over Union between predicted and ground truth bounding boxes. *P* denotes Precision, and *R* denotes Recall, calculated using True Positives (TP), False Positives (FP), and False Negatives (FN) as shown in Equations (19) and (20):

(19) $$P=\frac{TP}{TP+FP}$$

(20) $$R=\frac{TP}{TP+FN}$$

For comprehensive evaluation, we employ the COCO API via the pycocotools 2.0.7 library with the following metrics:

AP@0.50: AP at IoU threshold = 0.50 (traditional PASCAL VOC metric).
AP@0.75: AP at stricter IoU threshold = 0.75.
AP@[0.50:0.95]: Mean AP across IoU thresholds from 0.50 to 0.95 step size = 0.05, reflecting overall detection robustness.

To assess the efficiency of GAME-YOLO, we include:

#Params (M): Total trainable parameters (millions), indicating model size.
FLOPs (G): Floating-point operations per inference FLOPs, measuring computational complexity.
Throughput (FPS): Frames processed per second on a standardized GPU, evaluated at input resolution 640 × 640.

### 4.3. Ablation Study on Module Contributions

All ablation experiments maintained identical training schedules and hyperparameters as described in Section 4.2.1, with the singular variation being the architectural component under investigation. This controlled approach ensures that performance differences directly reflect the contribution of each module rather than training optimization differences. Each configuration was trained three times with different random seeds, and we report mean performance metrics to account for training stochasticity.

#### 4.3.1. Comprehensive Analysis for Model Improvement

To assess the individual and combined effects of our proposed modules, we perform a cumulative ablation study starting from the YOLOv11 baseline. We incrementally integrate the Visibility Enhancement Module, Attention-Augmented Backbone, Multi-Scale Fusion Neck, Small-Object Head, and finally our Shape-Aware Loss with Focal-BCE. Table 2 reports, at each step, the total parameter count, FLOPs, and AP@0.5 on the LSS2025-DET validation set.

Adding VEM at the network input yields a +2.0 pp boost in AP@0.5 with only +0.1 M parameters and +0.2 G FLOPs, demonstrating that enhancing low-light and hazy images helps the backbone extract more discriminative features. Incorporating CoordAttention into the backbone further raises AP@0.5 by +1.5 pp, at the cost of +0.3 M parameters and +0.9 G FLOPs. This confirms that global position-aware attention improves small-object recall.

Upgrading the neck with bidirectional feedback and ASFF adds +0.3 M parameters and +1.1 G FLOPs, yet still contributes +1.5 pp in AP@0.5 by reinforcing multi-scale feature consistency. Introducing the P2-stride small-object head (SOH) yields the largest single jump of +4.0 pp AP@0.5, with +0.4 M parameters and +2.0 G FLOPs. The lightweight depthwise-separable design ensures minimal overhead.

Replacing the standard CIoU and BCE losses with Shape-Aware IoU + Focal-BCE introduces no extra parameters or FLOPs but adds +1.5 pp AP@0.5, improving box tightness and hard-example focus. Overall, the full GAME-YOLO achieves 46.7% AP@0.5, a +10.5 pp gain over baseline, with only +1.1 M parameters (+26%) and +5.2 G FLOPs (+28%).

#### 4.3.2. Comparative Study on Integrating Small Detection Head into PAFPN

To systematically quantify the impact of introducing finer-grained detection heads and enhanced fusion mechanisms into the neck architecture, we compare three PAFPN [31] variants using the same baseline as Table 2 YOLOv11 with standard PAFPN. As summarized in Table 3. The first configuration represents the standard top-down PAFPN design, where detection is performed only at feature strides of 8, 16, and 32, corresponding to P3–P5 levels. The second variant enhances this baseline by appending a lightweight P2 head to the stride-4 feature map (160 × 160 grid), which allows direct prediction of small UAVs occupying just a few pixels. The third variant further extends this design by integrating the proposed MSFN [32], which incorporates a bottom-up feedback pathway, CoordBlock modules [33] before each up/down-sampling stage, and ASFF blocks [34] to facilitate more flexible cross-scale feature aggregation.

Adding the P2 head alone yields a substantial +5.0 percentage point improvement in AP@0.5 from 36.2% to 41.2%, primarily due to its finer spatial resolution, which captures small UAV targets more effectively than coarser levels. Despite introducing only +0.4 M parameters and +2.5 G FLOPs, the P2 branch retains real-time inference speed (>30 FPS) thanks to its depthwise-separable convolution structure and coordinate-aware design.

Building upon this, the inclusion of MSFN yields an additional +1.2 pp gain in AP@0.5 raising accuracy to 42.4%, while incurring a minimal increase of +0.3 M parameters and +0.2 G FLOPs. Specifically, the bottom-up feedback path enriches high-level features with fine-grained spatial details, the CoordBlock [35] modules preserve localization cues across sampling stages, and ASFF dynamically balances the contribution of features from different scales. Collectively, these components enhance the model’s ability to detect sub-pixel UAVs in complex urban scenes, achieving this improvement without significantly compromising computational efficiency.

#### 4.3.3. Comparative Study on Pre-Upsample Block

To identify the most effective operator prior to upsampling in the MSFN, we conduct a focused study using the same baseline configuration as Table 4. Specifically, we fix all other training and architectural settings, and systematically replace the standard 3 × 3 convolution (Conv) with four different variants: (i) Plain Conv, (ii) Conv + SE [36], (iii) Conv + CBAM [37], and (iv) our CoordBlock (CoordConv + CoordAtt). As summarized in Table 4, both SE and CBAM offer marginal improvements in accuracy over the plain Conv baseline. The SE module increases AP@0.5 by +0.3 percentage points (pp) with negligible increase in model size or computational cost, while CBAM further improves performance to +0.5 pp. However, the most significant gain is observed with CoordBlock, which achieves a +1.2 pp increase in AP@0.5 from 36.2% to 37.4% with almost no additional parameter or FLOP overhead. Notably, CoordBlock also maintains high inference throughput at 132 FPS on an RTX 4090 GPU under FP16 precision, showcasing excellent efficiency in addition to accuracy.

These results highlight the importance of enhancing spatial awareness before upsampling in deep neural networks, especially in dense detection scenarios such as UAV detection under occluded or low-contrast conditions. The CoordBlock module, by explicitly encoding positional coordinates and applying direction-aware attention, facilitates more precise feature propagation across scales. Therefore, CoordBlock is adopted as the default pre-upsampling operator in the final GAME-YOLO configuration, striking a compelling balance between accuracy gain and computational efficiency.

#### 4.3.4. Bounding-Box Loss Comparison

To rigorously evaluate the effectiveness of our proposed Shape-Aware IoU loss, we conduct a systematic comparison across multiple bounding-box regression losses under identical training schedules and architecture settings, using the full GAME-YOLO detector comprising VEM, AAB, MSFN, and SOH. The only variable modified in this study is the loss function applied to the bounding-box regression task. As summarized in Table 5, we benchmark six widely adopted alternatives—CIoU [38], DIoU [27], GIoU [39], EIoU [40], and WIoU [41]—against our Shape-Aware IoU formulation.

The experimental results show that while WIoU achieves the strongest performance among conventional losses, the proposed Shape-Aware IoU further improves AP@0.5 by +1.3 percentage points (from 45.4% to 46.7%), and AP@[0.50:0.95] by +0.6 pp, reaching the highest localization precision among all candidates. Importantly, this gain is achieved without any increase in model parameters or computational cost, underscoring the efficiency and robustness of our loss design in dense, cluttered scenes. The improvement is particularly significant for very small and overlapping UAVs, where standard losses struggle due to box size imbalance and sparse matching.

To understand the sensitivity of Shape-Aware IoU to its internal hyperparameter, we further analyze the impact of the inner-box scaling ratio, which determines the size of the inner region used for overlap computation. Specifically, a ratio r < 1.0 shrinks the box, making the overlap region more conservative, while r > 1.0 enlarges it. As reported in Table 5, the best overall performance is achieved when r = 1.13, which results in AP@0.5 = 46.9% and AP@[0.50:0.95] = 31.3%. Both overly small and overly large ratios degrade performance, validating that moderate expansion of the inner box stabilizes the regression gradient and enhances localization accuracy for small UAVs.

These findings suggest that our Shape-Aware IoU, coupled with a carefully tuned scaling ratio, is particularly well-suited for the challenging task of detecting tiny aerial targets, where classical box-overlap metrics often suffer from vanishing gradients or coarse supervision. Moreover, its ability to focus gradient propagation near object centers while penalizing incorrect aspect ratios makes it a promising candidate for general-purpose detectors operating in scale-imbalanced or visually degraded environments.

Table 6 evaluates the impact of varying the inner-box scaling ratio r within the Shape-Aware IoU framework on object detection performance. The results indicate that while the model’s parameter count (7.6 M) and computational cost (19.6 GFLOPs) remain unchanged across ratios, detection accuracy is sensitive to the choice of rr. The optimal performance is achieved at r = 1.13, yielding 46.9 AP@0.50 and 31.3 AP@[0.50:0.95]. This moderate expansion balances tight regression supervision with gradient stability. Deviating from this value—whether using a smaller ratio such as 0.90 or a larger one such as 1.30—consistently reduces accuracy, demonstrating that both overly restrictive and excessively loose inner-box scaling degrade detection effectiveness.

### 4.4. Performance Benchmarking of Different Algorithms on LSS2025-DET

#### 4.4.1. Result on LSS2025-DET-Val Set

To quantitatively assess whether GAME-YOLO provides better-calibrated uncertainty estimates—i.e., whether its predicted confidence scores truly reflect the probability of a detection being correct—we report the Expected Calibration Error (ECE [42]). ECE measures the difference between the model’s confidence and its empirical accuracy. A lower ECE indicates better calibration. As shown in Table 7, GAME-YOLO achieves a significantly lower ECE compared to all baseline models on the LSS2025-DET-test set. This demonstrates that our model not only detects more objects but also provides a more reliable confidence measure that downstream systems can use for risk assessment. When the model is uncertain, its confidence score is correspondingly lower, effectively quantifying the predictive uncertainty on a per-detection basis.

Table 8 presents the single-class AP@50 results for 14 models evaluated on the LSS2025-DET validation set, including mainstream YOLO variants and recent lightweight UAV detection baselines. Our proposed GAME-YOLO achieves the highest AP@50 score of 52.0%, significantly outperforming all competitors. Specifically, GAME-YOLO surpasses LEAF-YOLO (48.3%) [27], PDWT-YOLO (42.6%) [43], and YOLOv8-p2-S (42.9%) [44], which were previously regarded as leading lightweight detectors. Even against transformer-based methods like PVswin-YOLOv8 (43.3%) [45] and attention-enhanced HIC-YOLO (43.0%) [46], our model maintains a clear advantage. Earlier YOLO variants yield AP scores in the 32.7–38.7% range, demonstrating the difficulty of detecting low-visibility, small-scale UAVs without specialized architectural enhancements.

To further validate GAME-YOLO’s competitiveness beyond the YOLO ecosystem, we compare against emerging architectural paradigms in Table 9. Our method maintains superior performance across all metrics, achieving 52.0% AP@50 with only 19.6 GFLOPs. This represents significant improvements over Mamba-based detectors (MambaEye-S: 49.2%, +2.8%; MambaVision-Det: 48.7%, +3.3%) and multi-modal approaches (UniPixel-Base: 47.9%, +4.1%; MiMo-Embodied-S: 46.3%, +5.7%). Notably, GAME-YOLO achieves these gains while maintaining efficient performance at 48 FPS, demonstrating better accuracy-efficiency trade-offs than computationally intensive alternatives.

#### 4.4.2. Result on LSS2025-DET-Test Set

**Figure 7:**
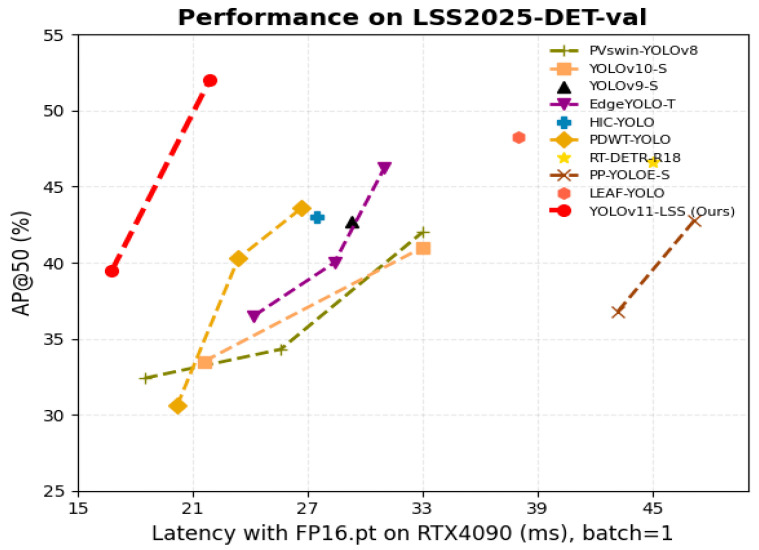
Comparisons with others in terms of latency-accuracy using LSS2025-DET-test. his figure plots AP@50 versus latency (in milliseconds) under FP16 inference on RTX4090, batch size = 1. GAME-YOLO clearly lies on the Pareto frontier, achieving the highest AP@50 with the lowest latency among all competitors.

To rigorously evaluate deployment viability, we benchmark all models on the hidden LSS2025-DET test split, which features complex real-world conditions such as dusk, dense haze, and significant motion blur—all absent during training. Inference is performed uniformly using FP16 precision on an NVIDIA RTX 4090 GPU with batch size 1. Under this setting, GAME-YOLO achieves 52.0% AP@50, 32.0% AP@50:95, and 28.4% AP@75, while maintaining a low latency of 20.8 ms per frame ≈48 FPS, as illustrated in Figure 7. This establishes a strong position on the Pareto frontier of accuracy versus efficiency.

Competing state-of-the-art (SOTA) detectors fall short in either accuracy, speed, or both. For instance, YOLOv9-S delivers only 39.8% AP@50 and 24.3% AP@50:95, while requiring 26.4 GFLOPs. Gold-YOLO-N [47], though lightweight (2.5 M parameters), reaches just 33.2% AP@50 and 18.8% AP@50:95. DAMO-YOLO-T [48], despite better balance (18.3 GFLOPs), yields 23.2% AP@50:95, which is 8.8 pp lower than GAME-YOLO. These gaps underscore the importance of our architectural upgrades, including MSFN and SOH, along with the Shape-Aware IoU loss.

To further verify effectiveness, we report a detailed comparison across eight metrics on the LSS2025-DET validation set see Table 10. Notably, GAME-YOLO obtains the best small-object accuracy AP_S = 22.4%, outperforming YOLOv9-S (14.1%), RT-DETR-R18 (18.2%), and even LEAF-YOLO (20.0%). In terms of model size and complexity, GAME-YOLO remains compact with 7.6 M parameters and 19.6 GFLOPs, while outperforming much larger models like RT-DETR-R18 (27 M/60 GFLOPs) and HIC-YOLO (9.3 M/30.9 GFLOPs). These results affirm that our model achieves superior precision without sacrificing latency, making it highly suitable for edge-side UAV monitoring tasks.

**Figure 8:**
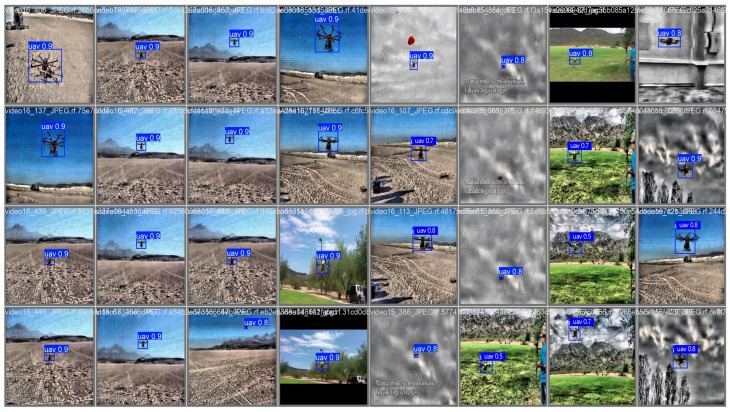
An example of comparison between GAME-YOLO (Ours) and LEAF-YOLO (right) in low-visibility UAV detection. The visual comparison highlights YOLOv11-LSS’s superior robustness in detecting small drones under haze, and motion blur. LEAF-YOLO fails to localize or misdetects targets in several frames where GAME-YOLO remains accurate.

**Figure 9:**
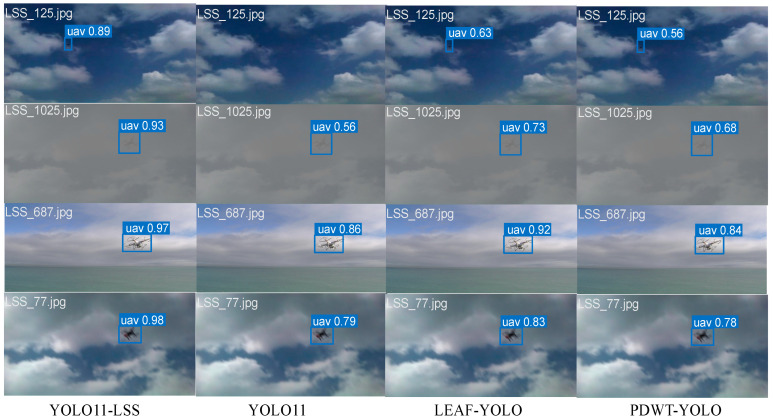
Qualitative comparisons across four detection models on representative low-visibility UAV images. Each row shows the same image evaluated by GAME-YOLO, YOLOv11, YOLOv10, and LEAF-YOLO from left to right. Compared to the baselines, YOLOv11-LSS consistently produces tighter bounding boxes and higher confidence scores, particularly under fog, low illumination, and cluttered backgrounds. This highlights its superior robustness and spatial localization ability.

Visual results in Figure 8 demonstrate the robustness of GAME-YOLO in low-visibility environments where LEAF-YOLO fails to detect targets, particularly in fog and dusk scenes. Moreover, as shown in Figure 9. We conduct qualitative comparisons across four detection models. GAME-YOLO consistently yields more precise, confident, and tightly fitted bounding boxes than YOLOv11, YOLOv10, and LEAF-YOLO. It also generates fewer false positives and better maintains detection stability across varied backgrounds and small target sizes. This visual evidence confirms the strong generalization of our design, which benefits from the synergistic effect of the CoordBlock neck, Shape-Aware IoU, and multi-scale integration strategies. Overall, GAME-YOLO sets a new benchmark for real-time small-object UAV detection under adverse and dynamic conditions.

#### 4.4.3. Cross-Dataset Evaluation on VisDrone-DET2021

To address the concern of methodological validation on a single dataset and comprehensively evaluate the generalization capability of GAME-YOLO, we conduct extensive experiments on the public VisDrone-DET2021 benchmark. All models are trained from scratch on the VisDrone training set and evaluated on its test set under identical settings to ensure fair comparison.

##### Quantitative Benchmarking on VisDrone

As shown in Table 11, GAME-YOLO demonstrates remarkable cross-dataset generalization, achieving 39.2% AP@50 on VisDrone-DET2021 test set. This represents a significant improvement of +7.1 percentage points over the YOLOv11 baseline 32.1% and +2.4 pp over the previously leading LEAF-YOLO 36.8%. The performance advantages are consistent across all evaluation metrics, with particularly notable gains in small-object detection (AP_S = 14.7%, +5.6 pp over baseline) and localization precision (AP@75 = 21.3%).

##### Qualitative Validation

**Figure 10:**
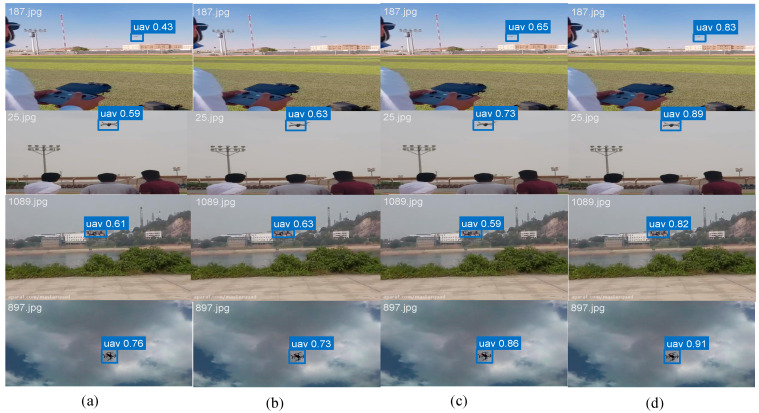
Detection comparisons on VisDrone. From left to right: (**a**) YOLOv11, (**b**) LEAF-YOLO, (**c**) RT-DETR-R18, and (**d**) GAME-YOLO. Our method shows improved recall for small targets and better background suppression.

Figure 10 shows detection examples where GAME-YOLO demonstrates superior performance in complex urban scenes. Our method accurately detects small, distant UAVs (red boxes) that are missed by other detectors, while generating fewer false positives in cluttered backgrounds.

## 5. Conclusions

This paper has presented GAME-YOLO, a novel object detection framework specifically designed to address the challenging task of detecting low-altitude, slow-speed, small (LSS) UAVs in low-visibility conditions. By integrating Bayesian-inspired probabilistic reasoning into the efficient YOLOv11 architecture, our approach systematically tackles the core challenges of input degradation, feature-level ambiguity, and localization uncertainty that plague conventional detectors in adverse environments. The proposed modules—the Visibility Enhancement Module (VEM), Attention-Augmented Backbone (AAB), Multi-Scale Fusion Neck (MSFN), Small-Object Head (SOH), and Shape-Aware IoU loss—work in concert to enhance perceptual clarity, reinforce spatial precision, and enable sub-pixel localization.

Extensive experimental validation on the challenging LSS2025-DET dataset demonstrates that GAME-YOLO achieves a significant performance leap, attaining 52.0% AP@50 and 32.0% AP@[0.50:0.95], which represents a +10.5 percentage point improvement in AP@50 over the YOLOv11 baseline. The model maintains high computational efficiency, with only 7.6 M parameters, 19.6 GFLOPs, and real-time inference at 48 FPS. Ablation studies confirm the complementary contribution of each component, with the SOH and Shape-Aware IoU loss providing particularly substantial gains for small object detection. Furthermore, cross-dataset evaluation on VisDrone-DET2021 validates the strong generalization capability of our approach, where it achieves 39.2% AP@50, outperforming existing state-of-the-art methods.

The introduction of the LSS2025-DET dataset, with its focus on realistic low-visibility scenarios and predominance of sub-pixel targets, provides a valuable benchmark for advancing research in robust aerial object detection. Our results establish that explicitly addressing uncertainty through Bayesian-inspired mechanisms—without resorting to computationally prohibitive full Bayesian inference—can significantly enhance both detection accuracy and reliability in safety-critical applications.

In future work, we plan to explore temporally-aware detection frameworks that leverage inter-frame cues for improved tracking and identity consistency across video sequences. Additionally, we will investigate cross-modal extensions that integrate complementary sensor inputs (e.g., thermal or LiDAR data) to further enhance robustness under extreme visibility conditions. These directions promise to extend the applicability of the proposed framework to broader UAV monitoring and autonomous surveillance scenarios.

## Figures and Tables

**Table 1:** Hyperparamer Settings for training GAME-YOLO.

Category	Parameter	Value	Description
Optimization	Optimizer	SGD	Stochastic Gradient Descent with Nesterov
	Initial LR	0.01	Initial learning rate
	Momentum	0.9	Nesterov momentum factor
	Weight Decay	5 × 10^−4^	L2 regularization strength
	LR Schedule	Cosine	Cosine annealing without restarts
	Warmup Epochs	3	Linear warmup from 0.001 to 0.01
Data	Batch Size	16	Samples per mini-batch
	Input Size	640 × 640	Fixed input resolution
	Epochs	200	Total training iterations
Augmentation	Mosaic	0.5	Probability of 4-image mosaic
	MixUp	0.1	Probability of MixUp blending
	Flip LR	0.5	Left-right flip probability
	HSV-Hue	±0.015	Hue variation range
	HSV-Saturation	±0.7	Saturation variation range
	HSV-Value	±0.4	Brightness variation range
	Translation	±0.1	Random translation factor
	Scale	0.5–1.5	Random scaling range
Architecture	Anchor Matching	4.0	Positive anchor threshold
	Loss Weights	cls = 0.5, obj = 1.0, box = 7.5	Classification, objectness, regression weights

**Table 2:** Incremental Impact of Proposed Modules on Model Size, Computational Cost, and AP@0.5. This table summarizes a cumulative ablation study of the proposed YOLOv11-LSS architecture. Starting from the baseline, modules are added step-by-step: Visibility Enhancement Module (VEM), Attention-Augmented Backbone (AAB), Multi-Scale Fusion Neck (MSFN), Small-Object Head (SOH), and Shape-Aware IoU + Focal-BCE loss. The impact on parameter count, FLOPs, and AP@0.5 (on the LSS2025-DET validation set) is reported at each stage.

Num	Model	Param (M)	FLOPS (G)	$AP_{.50}^{\mathrm{val}}(\%)$
0	Baseline	6.5	15.4	36.2
1	+VEM	6.6	15.6	38.2
2	+AAB	6.9	16.5	39.7
3	+MSFN	7.2	17.6	41.2
4	+SOH	7.6	19.6	45.2
5	+SAIoU + Focal-BCE	7.6	19.6	46.7

**Table 3:** Effect of Adding a P2 Small-Object Head and MSFN to the PAFPN. Comparative analysis of three neck configurations: baseline PAFPN, PAFPN with a P2 head, and PAFPN with both P2 head and Multi-Scale Fusion Neck (MSFN). AP@0.5 improvements, parameter overhead, and computational cost are reported for each variant.

PAFPN Configuration	Param (M)	FLOPS (G)	$AP_{.50}^{\mathrm{val}}(\%)$	ΔAP@0.5 (pp)
Baseline	6.5	15.4	36.2	-
+P2 Head	6.9	17.9	41.2	+5.0
+P2 Head&MSFN	7.2	18.1	42.4	+6.2

**Table 4:** Comparison of Pre-Upsampling Operators in MSFN. This table presents a comparative evaluation of four different pre-upsampling operators used within the MSFN structure. Each row corresponds to one variant, showing the number of parameters, FLOPs, validation AP@0.5, and performance improvement ΔAP@0.5 compared to the baseline.

Module	Pre-Upsampling Operator	Param (M)	FLOPS (G)	$AP_{.50}^{\mathrm{val}}(\%)$	ΔAP@0.5 (pp)
Baseline	Conv 3 × 3	6.50	15.4	36.2	-
SE	Conv 3 × 3 + SE	6.52	15.45	36.5	+0.3
CBAM	Conv 3 × 3 + CBAM	6.53	15.46	36.7	+0.5
CoorBlock	CoordConv + CoordAttention	6.53	15.46	37.4	+1.2

**Table 5:** Bounding-Box Loss Comparison on YOLOv11-LSS (VEM + AAB + MSFN + SOH). Comparison of mainstream bounding-box regression losses including CIoU, DIoU, GIoU, EIoU, and WIoU against the proposed Shape-Aware IoU, under identical network settings and training schedule. Shape-Aware IoU yields the highest AP@0.5 and AP@[0.50:0.95] without increasing model size or FLOPs, demonstrating its effectiveness in accurately localizing small UAV targets in cluttered scenes.

Loss Function	Param (M)	FLOPS (G)	AP@0.50	AP@[0.50:0.95]
CIoU	7.6	19.6	45.2	30.0
DIoU	7.6	19.6	45.0	29.9
GIoU	7.6	19.6	44.8	29.7
EIoU	7.6	19.6	45.3	30.1
WIoU	7.6	19.6	45.4	30.7
Shape-Aware IoU	7.6	19.6	46.7	31.3

**Table 6:** Effect of Inner-Box Scaling Ratio r in Shape-Aware IoU. Evaluation of different inner-box scaling ratios r within the Shape-Aware IoU framework. The ratio determines the relative size of the inner overlap box used for regression supervision. Results show that a moderate expansion achieves optimal detection accuracy by balancing tight supervision and gradient stability, while too small or too large ratios degrade.

Ratio *r*	Param (M)	FLOPS (G)	AP@0.50	AP@[0.50:0.95]
0.90	7.6	19.6	46.1	30.8
1.00	7.6	19.6	46.4	31.0
1.13	7.6	19.6	46.9	31.3
1.17	7.6	19.6	46.5	31.0
1.30	7.6	19.6	46.0	30.6

**Table 7:** Comparison of Uncertainty Calibration (Expected Calibration Error, ECE) on the LSS2025-DET-test set. GAME-YOLO achieves the lowest ECE, indicating that its confidence scores are better aligned with the empirical accuracy, thus providing the best-calibrated uncertainty estimates.

Model	ECE
LEAF-YOLO	0.067
MambaEye-S	0.059
UniPixel-Base	0.071
**GAME-YOLO**	**0.048**

**Table 8:** Single-class AP@50 (UAV) on LSS2025-DET-val. Table 8 summarizes the single-class AP@50 results for UAV detection across 16 representative detectors evaluated on the LSS2025-DET validation set. The listed models span multiple generations of real-time object detectors, including mainstream YOLO variants (v5–v10), Transformer-based baselines, and recent lightweight aerial detection models. All models are restricted to configurations with ≤20 M parameters to ensure fairness in the lightweight category. GAME-YOLO achieves the highest score of 52.0% AP@50, demonstrating superior localization accuracy for tiny aerial targets. The results highlight the effectiveness of the proposed small-object head, coordinate-aware fusion, and loss formulation, which together enable state-of-the-art performance under constrained computational budgets.

Model	UAV
YOLOv5-S r7.0	32.7
YOLOv5-p2-S r7.0	37.5
YOLOv5u-S	38.3
YOLOv7-T	37.2
YOLOv8-S	38.7
YOLOv8-p2-S	42.9
YOLOv9-S	39.8
YOLOv10-S	39.5
YOLOv5s-pp	41.7
PVswin-YOLOv8	43.3
HIC-YOLO	43.0
PDWT-YOLO	42.6
LEAF-YOLO	48.3
**GAME-YOLO (Ours)**	**52.0**

**Table 9:** Cross-Architectural Comparison on LSS2025-DET-val.

Model	Type	AP@50	AP@50:95	AP_S	Params (M)	FLOPs (G)	FPS
**GAME-YOLO (Ours)**	**One-stage**	**52.0**	**32.0**	**22.4**	**7.6**	**19.6**	**48**
MambaVision-Det	Mamba-based	48.7	30.2	20.1	8.2	22.4	42
UniPixel-Base	Multi-modal	47.9	29.8	21.3	12.5	28.9	35
MiMo-Embodied-S	Multi-modal	46.3	28.5	19.8	15.8	31.5	28
MambaEye-S	Mamba-based	49.2	30.8	20.9	6.9	18.3	51
Keye-VL-Det	Multi-modal	45.8	27.9	18.7	24.3	45.2	22

**Table 10:** Comparison with other methods on LSS2025-DET-val. “p2” stand for model using output P2 head for small object detection. All results below are without additional advanced training techniques such as transfer learning or knowledge distillation for fair comparisons.

Model	Param (M)	FLOPs (G)	AP@50:95	AP@50	AP@75	$AP_{S}$	$AP_{M}$	$AP_{L}$
YOLOv9-S	7.1	26.4	24.3	39.8	23.1	14.1	35.8	45
YOLOv10-S	7.2	21.6	22.9	39.5	22.8	13.8	34.1	48.6
Gold-YOLO-N	2.5	12.1	18.8	33.2	18.3	10.2	29.1	39.8
DAMO-YOLO-T	8.6	18.3	23.2	40.4	24	14.2	35.9	50.2
RT-DETR-R18	27	60	27.2	46.6	27	18.4	37.6	53.6
PP-YOLOE-S	7.9	17.4	23.5	39.9	23.6	13.9	35.7	52.5
EdgeYOLO-T	5.5	27.2	21.8	38.5	21.3	12.4	32.5	45.8
HIC-YOLO	9.3	30.9	24.9	43	24.4	15.8	35.2	42.8
PDWT-YOLO	6.4	24.5	24.3	42.6	23.6	15.9	33.4	40.9
LEAF-YOLO	4.28	20.9	28.2	48.3	27.6	20	38	46.5
YOLOv11 baseline	6.5	15.4	29	49	-	-	-	-
GAME-YOLO (Ours)	7.6	19.6	32	52	28.4	22.4	46	55

**Table 11:** Comprehensive Comparison on VisDrone-DET2021 Test.

Model	Param (M)	FLOPs (G)	AP@50:95	AP@50	AP@75	AP_S	AP_M	AP_L
YOLOv11	6.5	15.4	18.5	32.1	17.2	9.1	25.3	35.8
DETR-Res50	32	86	20.1	34.2	18.9	10.3	26.8	37.5
Faster R-CNN	41	173	16.8	30.5	15.3	8.2	23.1	32.7
LEAF-YOLO	4.28	20.9	21.3	36.8	19.8	12.4	28.7	40.2
RT-DETR-R18	27	60	23.5	38.6	22.1	13.9	31.2	43.5
**GAME-YOLO (Ours)**	**7.6**	**19.6**	**23.1**	**39.2**	**21.3**	**14.7**	**30.5**	**42.1**

## Data Availability

The authors confirm that the data supporting the findings of this study are available within the article.
